# Reuse of Red Mud and Bauxite Tailings Mud as Subgrade Materials from the Perspective of Mechanical Properties

**DOI:** 10.3390/ma15031123

**Published:** 2022-01-31

**Authors:** Xiaoduo Ou, Shengjin Chen, Jie Jiang, Jinxi Qin, Lu Zhang

**Affiliations:** 1 College of Civil Engineering and Architecture, Guangxi University, Nanning 530004, China; ouxiaoduo@163.com (X.O.); 1810401003@st.gxu.edu.cn (S.C.); 2 Guangxi Hualan Geotechnical Engineering Co., Ltd., Nanning 530004, China; 3 Guangxi Xinfazhan Communication Group Co., Ltd., Nanning 530029, China; qinjinxi1995@163.com; 4 College of Civil Engineering and Architecture, Guilin University of Technology, Guilin 541004, China

**Keywords:** Bayer red mud, bauxite tailings mud, subgrade material, mechanical properties, environmental protection

## Abstract

In order to reuse red mud and bauxite tailings mud (two typical aluminum industrial wastes) to reduce the occupation of land resources and environmental damage, these two wastes were combined to develop subgrade materials for the first time. With different combinations, the effects of the amounts of red mud, tailings mud, and cementitious materials on the strength of tested subgrade materials were investigated. The mechanism of strength growth was analyzed by a micro-test. The test results showed that the material strength of three combinations met the requirements when the unconfined compression strength (UCS) of all combinations increased with age. The UCS of the A_1_BC_2_ combination (the mass ratio of red mud and tailings mud was 2:1, the mass ratio of cement and quicklime was 1:1, and the mass ratio of waste and cementitious materials was 1:0.2) was the best, with the UCS being 3.03 MPa in 7 days. Microscopic imaging showed that specimens with high red mud contents had compact structures without cracks. The strength of these materials is mainly due to hydration reactions and pozzolanic reactions; the cementitious products generated by the reactions solidify Na^+^ and inhibit the release of OH^−^, while the addition of tailings mud can reduce the content of Na_2_O in the material, which makes the environmental compatibility of the A_3_BC_2_ combination the best (the mass ratio of red mud and tailings mud was 1:2, the mass ratio of cement and quicklime was 1:1, and the mass ratio of waste and cementitious materials was 1:0.2). Its pH value was 8.75. This experiment verifies the feasibility of the combined application of red mud and tailings mud in subgrade materials. To this end, a feasible scheme for the simultaneous consumption of these two kinds of aluminum industrial wastes has been proposed.

## 1. Introduction

Aluminum is one of the most widely used nonferrous metallic materials in the world. In the production of aluminum, two wastes are generated in large volumes: red mud and bauxite tailings mud. Red mud is an alkaline solid waste, and tailings mud is the mud waste produced by grinding and washing bauxite ore. These wastes have adverse impacts on the ecological environment. The utilization of red mud and tailings mud is very low, and the waste yards for such wastes occupy large areas of land [1]. In order to eliminate the adverse impacts of these wastes on land resources and the ecological environment, it is of significance to find an economical way to utilize large amounts of red mud and bauxite tailings mud, especially in subgrade engineering.

Over the past few decades, the research on bauxite tailings mud mainly focuses on its physical and mechanical properties [2,3], drainage mechanism [4,5,6] and consolidation characteristics [7]. There are few application studies on subgrade materials. However, due to the mechanical properties of clay soil, bauxite tailings mud can be used in the field of road engineering after reducing its water content.

Currently, the utilization of red mud mainly surrounds three aspects: extracting valuable metals from the red mud [8,9]; using red mud as an adsorbent or catalyst for environmental remediation and treatment [10,11,12]; and using red mud as a mineral raw material in the field of construction [13,14,15]. The application of red mud to subgrade materials is an embodiment of this third aspect [16]. The mechanical properties of pure red mud or a combination of red mud and other industrial wastes (such as fly ash, slag, desulfurization gypsum, etc.) can meet the application requirements of low-grade road subgrade materials under the condition of adding certain additives [16]. Sahoo and Mohanty (2016) studied the effectiveness of red mud as a subgrade material based on the engineering characteristics of red mud. It was found that when the proportion of red mud was 2.9, the optimal water content was 23.25%, the maximum dry density was 1.81 g/cm^3^, the liquid limit was 31.20%, the UCS was 0.191 MPa, and the CBR was 7.5% [17]. Li et al. (2021) found that when red mud, fly ash, and desulfurization gypsum were used to prepare road base materials, the unconfined compressive strength of road base materials met the strength requirements of highways, and the ion leaching concentration met drinking water standards [18]; Liu et al. (2018) used the lime-fly ash method to stabilize red mud and found that the main factor affecting UCS for seven days was the proportion of lime and ash followed by the chemical composition of Bayer RM and, finally, the amount of lime and fly ash [19].

Both bauxite tailings mud and red mud are stored in alumina production enterprises, which are both convenient and cost-effective to use. Combining bauxite tailings mud and red mud to develop subgrade materials can not only reuse those two wastes but also reduce environmental hazards.

Considering the feasibility of bauxite tailings mud and red mud as subgrade materials, this experiment aims to apply these two typical aluminum industrial wastes to the subgrade of low-grade roadways. This paper puts forward new research that can not only save the construction cost of subgrade engineering but also realize the reuse of waste and the sustainable development of the environment. The results provide an important guideline for the application of red mud and tailings mud to subgrade engineering.

## 2. Materials and Methods

### 2.1. Materials

**Figure 1:**
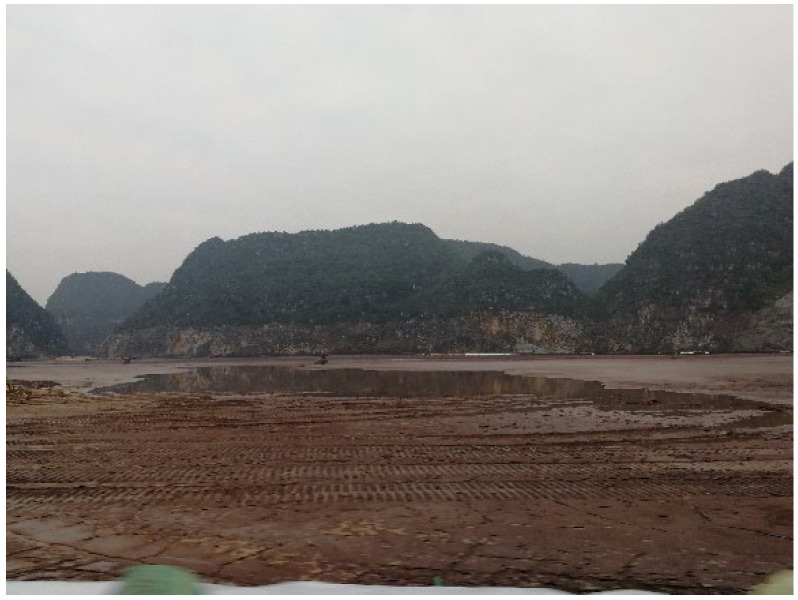
Photograph of the red mud reservoir.

**Figure 2:**
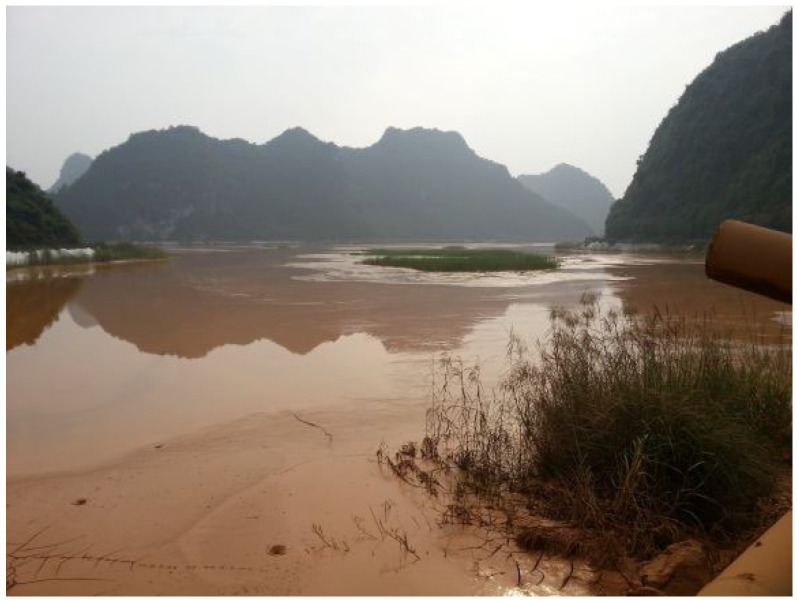
Photograph of the tailings mud reservoir.

The waste muds used in this study were mixed Bayer red mud and bauxite tailings mud, which were collected from Guangxi Xinfa Aluminum Electricity Co., Ltd., in Baise City, China (Figure 1 and Figure 2). The cementitious materials used in the test were commercial Portland cement (P.O42.5) and quicklime (CaO). The chemical compositions of red mud and tailings mud are listed in Table 1. It was noted that the red mud contains more Na_2_O, which can provide hydroxyls during the hydration process.

To prepare mixtures of the above-mentioned materials, three factors were mainly considered: the mass ratio of red mud to tailings mud (A), the mass ratio of cement to quicklime (B), and the mass ratio of waste to cementitious materials (C). To investigate the influences of these three factors, mixtures with different proportioning ratios were prepared. As listed in Table 2, for all mixtures, the mass ratio of cement and quicklime was fixed to be B = 1:1. Three mass ratios of red mud to tailings mud were prepared: A_1_ = 2:1, A_2_ = 1:1, and A_3_ = 1:2. For these mixtures, three mass ratios of waste material (red mud and tailings mud) and cementitious material were designed: C_1_ = 1:0.1, C_2_ = 1:0.2, and C3 = 1:0.3. With such a combination, 9 types of mixtures were made.

### 2.2. Specimen Preparation

The red mud and tailings mud were oven-dried. Then, the red mud, tailings mud, and quicklime were weighed according to the desired proportion ratios in Table 2 and mixed evenly. Afterwards, deionized water was sprayed into the mixed powder to prepare mixtures. The wetted mixtures were sealed in the curing room. After standing for 12 h, the cement was added to the pre-mixed materials. The well-prepared mixtures were subjected to subsequent mechanical tests (see Table 3 for more details). The subsequent test should be conducted within one hour after adding the cement.

### 2.3. Mechanical Tests

Compaction tests, unconfined compressive strength (UCS) tests, and California bearing ratio (CBR) tests were conducted to reveal the mechanical properties of the waste subgrade materials according to the standard test procedures stipulated in the Chinese Standard of JTG 3430-2020 [20]. The detailed test program is illustrated in Table 3.

**Figure 3:**
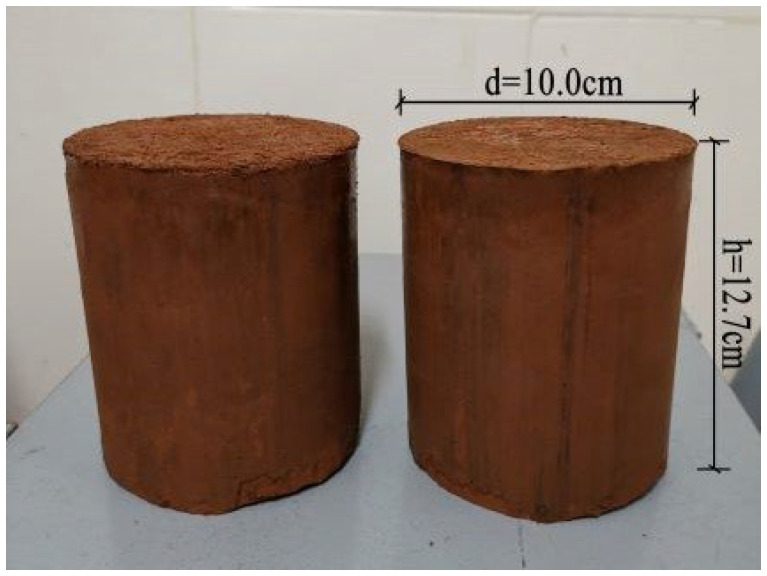
Specimens for the compaction test.

The compaction test was conducted with different initial water contents ranging from 17% to 40%. The detailed mixture is listed in Table 2. Based on the Chinese Standard JTG 3430-2020 [20], the specimens were compacted in 3 layers. Each layer was compacted for 27 strokes by a 4.5 kg hammer with a falling distance of 45 cm. The compacted specimens with diameters of 10 cm and heights of 12.7 cm (Figure 3) were removed from the center to determine their final water contents and dry densities.

**Figure 4:**
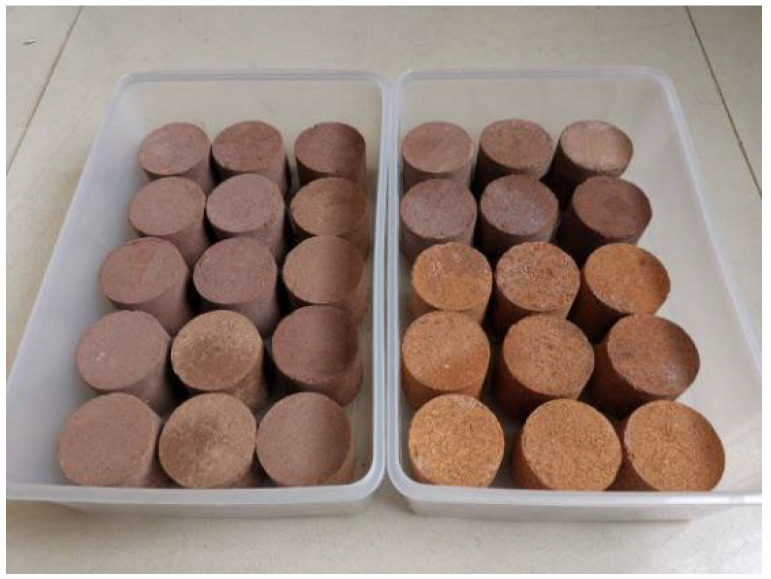
Specimens for the unconfined compression test.

The unconfined compression strength and CBR tests were conducted on compacted specimens with optimal water contents and were 94% compact. The well-prepared mixture was compacted to cylindrical columns 5 cm in height and 5 cm in diameter (Figure 4). After compaction, the specimens were placed under standard curing conditions with a controlled temperature of 20 °C and relative humidity of 95% for 1, 7, 28, and 60 days. For comparison, the additional unconfined compression tests were conducted on the BC_2_ combinations cured by immersing them in water for 28 days to verify the curing condition.

The CBR test was carried out on the A_3_BC_2_ combination after 7 days of curing (with the UCS exceeding 2 MPa).

### 2.4. SEM Tests

For further investigation of the growth of strength, micro-feature analysis was carried out on the specimens by using a scanning electron microscope (SEM) (S-3400N type) produced by Hitachi, in Tokyo, Japan, with magnification ranging between 20 and 300,000 times. The temperature of the specimen observation chamber was set to 50 °C, and the pressure was set to 650 Pa. Before the observations, the tested specimens were cut into cubic blocks with dimensions of 4 mm × 8 mm × 4 mm. The natural section is taken as the observation surface, and the back surface is pasted on the conductive adhesive. After spraying gold on the surface and side, the test and observations can be carried out.

### 2.5. pH Tests

Red mud and tailing mud are solid wastes from the aluminum industry. The only difference is whether alkali pollution exists or not in tailing mud. The impact of the release of alkali pollution on the environment needs to be considered in the process of its use. Therefore, according to the Chinese Standard GB7023-86 [21], the thunder magnetic pH meter (PHS-3C) was used to test the pH values of leach solution of specimens with 28 d curing ages at a room temperature of 25 °C, which was produced by Shanghai INESA Scientific Instrument Co., Ltd., in Shanghai, China.

## 3. Results and Discussion

### 3.1. Compaction Tests

**Figure 5:**
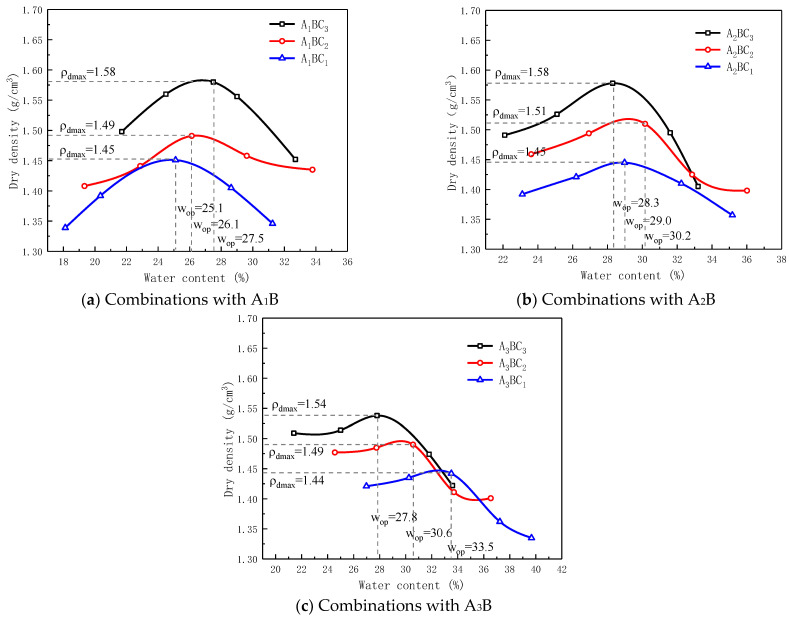
Relation between maximum dry density and optimum water content of various combinations (**a**–**c**).

The compaction curve of each group of specimens is shown in Figure 5. It shows that the optimum water content of each combination was between 25.1% and 33.5%, and the maximum dry density was between 1.44 g/cm^3^ and 1.58 g/cm^3^. The A_1_B combination was mainly composed of red mud waste. Herein, the optimal water content and maximum dry density increased with the increase in the cementitious material. The A_3_B combination mainly consisted of tailings mud waste. The optimum water content decreased with the increase in the cementitious material and the maximum dry density.

### 3.2. UCS Tests

**Figure 6:**
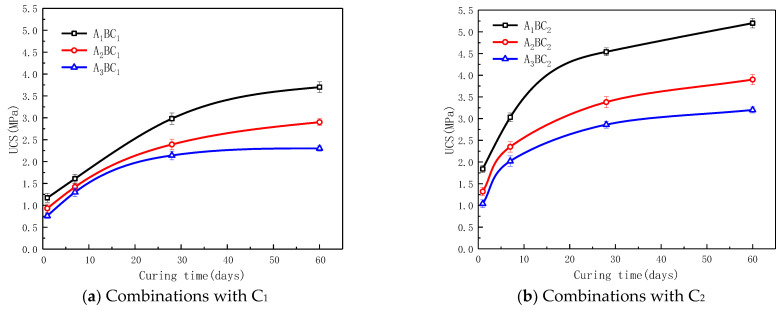

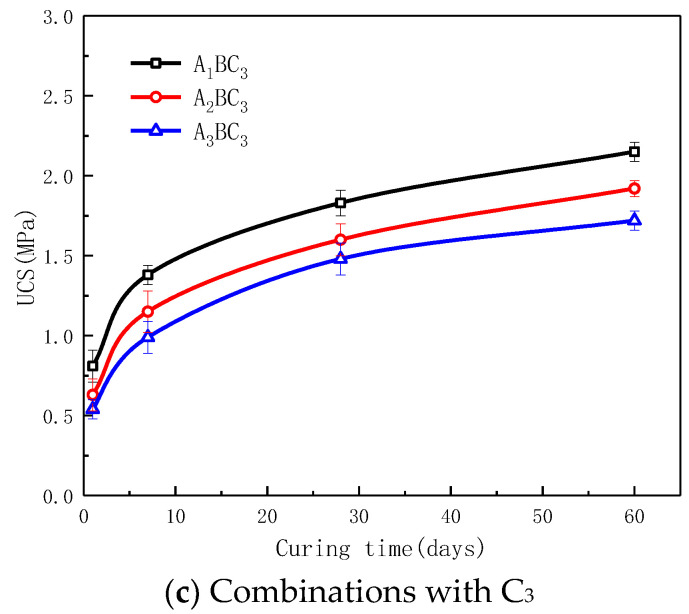
Change graphs of UCS of different combinations with the same dosage of cementitious material (**a**–**c**).

Figure 6 shows the UCS development of specimens with curing time. The UCS of all the combinations increased with age; however, the rate of increase in the strength of most combinations tends to be gentle after 28 days. This may be caused by the hydration reactions. With the aging process, the material strength increased; but when the ions such as Ca^2+^ and AlO_2_^−^ involved in the hydration reactions were consumed in large quantities, the rate of increase in the material strength started to slow.

Moreover, as shown in Figure 6b, the UCS of the C_2_ combinations presented in the descending order of A_1_BC_2_, A_2_BC_2_ and A_3_BC_2_; the UCS results of combinations with C_1_ and C_3_ showed a similar trend, see in Figure 6a,c. Upon further comparison of the strength increases due to the variation in the ratio of the wastes, it was observed that when the ratio of red mud to tailings mud increased from 1:1 (A_2_) to 2:1 (A_1_), the increase in UCS was higher than when the ratio of red mud to tailings mud increased from 1:2 (A_3_) to 1:1 (A_2_). This indicates that the red mud had a more positive effect on the strength of the material than the tailings mud since the red mud contains more Na_2_O. The strength was enhanced due to the increased production of hydroxyls from the hydration process.

**Figure 7:**
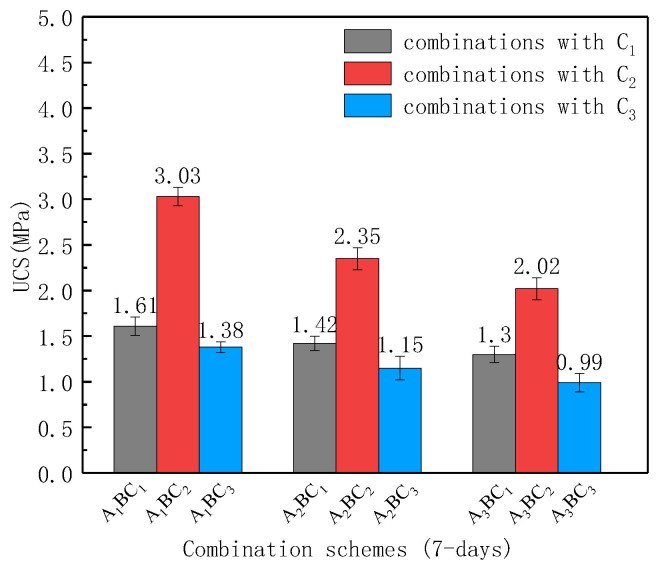
Comparisons of the UCS of each combination cured for 7 days.

**Figure 8:**
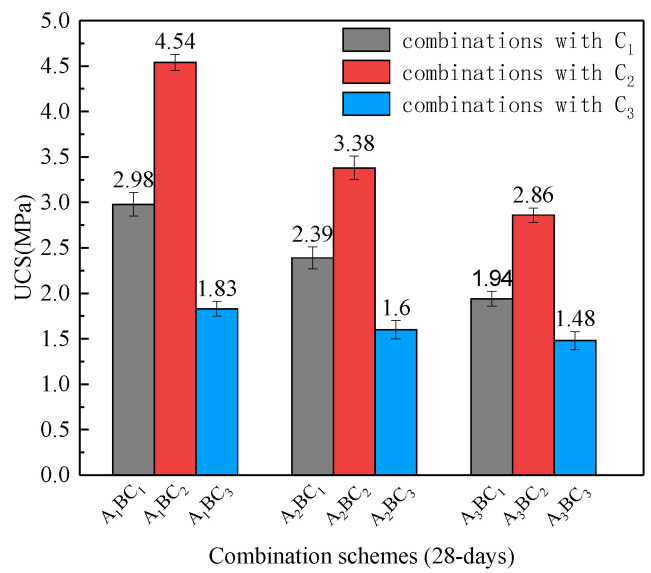
Comparisons of the UCS of each combination cured for 28 days.

Figure 7 and Figure 8 compare the UCS of the combinations cured for 7 days and 28 days. For the A_1_B combinations, the UCS of the A_1_BC_1_, A_1_BC_2_ and A_1_BC_3_ combinations were 1.61 MPa, 3.03 MPa, and 1.38 MPa, respectively. The UCS results from highest to lowest were A_1_BC_2_, A_1_BC_1_ and A_1_BC_3_, and those of the combinations of A_2_B and A_3_B showed a similar pattern. This reflects that, for a given ratio of the red mud and tailings mud, the relationship between the UCS of the specimen and the content of cementitious materials was not monotonous. Based on the results, it can be speculated that when C (the mass ratio of waste to cementitious materials) is 1:0.1, the hydration reaction between cementitious materials and waste materials is sufficient, but there are fewer hydration reaction products due to fewer cementitious materials. When C is 1:0.2, a similar amount of cementitious materials are produced so that the hydration reaction between cementitious materials and waste materials is sufficient; thus, the highest UCS strength was produced accordingly. However, when C is 1:0.3, the amount of cementitious material is excessive, resulting in carbonization after the hydration reaction [22], which reduces the strength instead. Therefore, the ratio of 1:0.2 (C_2_) is suggested as the optimum mass ratio of waste to cementitious materials. For this ratio, the UCS of the A_1_BC_2_, A_2_BC_2,_ and A_3_BC_2_ combinations cured for 7 d were larger than 2 MPa, meeting the requirements for the compressive strength of general, ordinary subgrade materials (UCS ≥ 2 MPa) based on the Chinese Standard JTG/T F20-2015 [23], and the A_1_BC_2_ combined strength was the highest.

**Figure 9:**
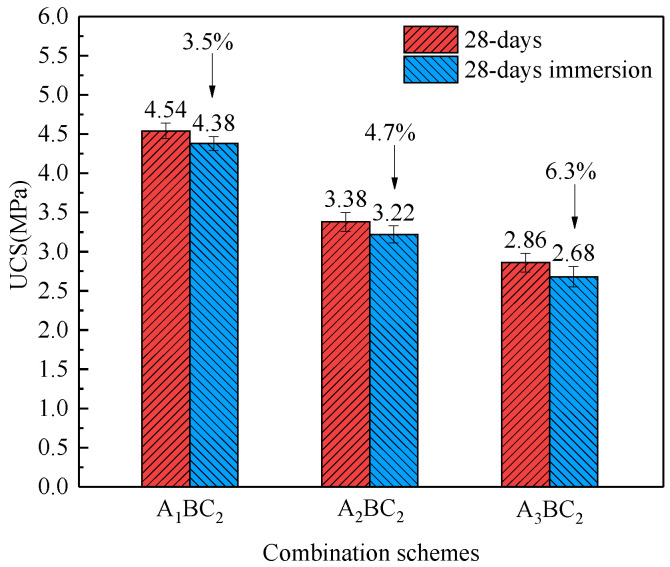
Comparisons of the UCS of BC_2_ composites cured for 28 days with and without immersion.

Figure 9 compares the UCS of the BC_2_ combinations cured with and without immersion for 28 days. The unconfined compression strengths of the A_1_BC_2_, A_2_BC_2_, and A_3_BC_2_ combinations cured without immersion were 4.54 MPa, 3.38 MPa, and 2.86 MPa, respectively; the corresponding values for combinations with immersion were 4.38 MPa, 3.22 MPa, and 2.68 MPa. Strength losses of 3.5%, 4.7%, and 6.3% due to immersion were obtained, creating an average of 4.9%. Though short-term immersion might soften a specimen, the strength of the immersed specimen still meets durability requirements. From a practical point of view, the influence of curing conditions on the strength of BC_2_ combinations is insignificant.

### 3.3. CBR Tests

The test results showed that the CBR value of the A_3_BC_2_ specimen reached 55.8%, which exceeded the minimum CBR requirement (6%) of the proposed subgrade material based on the Chinese Standard JTG D30-2015 [24]. The strength of the compacted composite material mainly depends on the material’s friction strength and the hydration products produced by the hydration reaction of the subgrade material. The CBR value obtained from the test was quite high, which is related to the bonding effect from the hydration reaction.

### 3.4. SEM Tests

**Figure 10:**
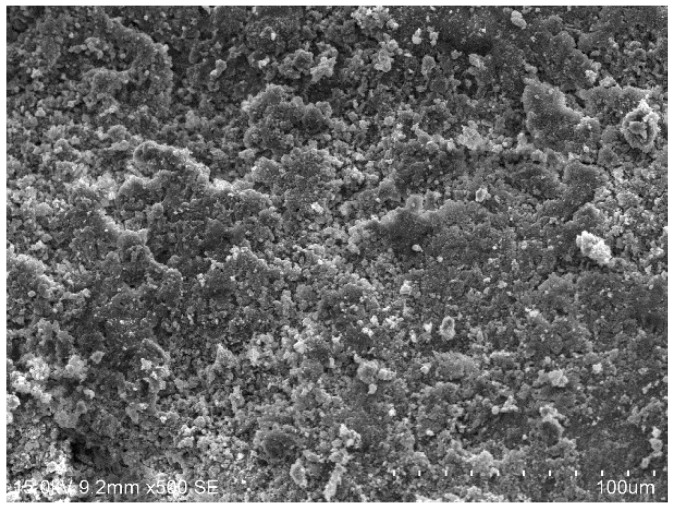
500× SEM image of the A_1_BC_2_ combination.

**Figure 11:**
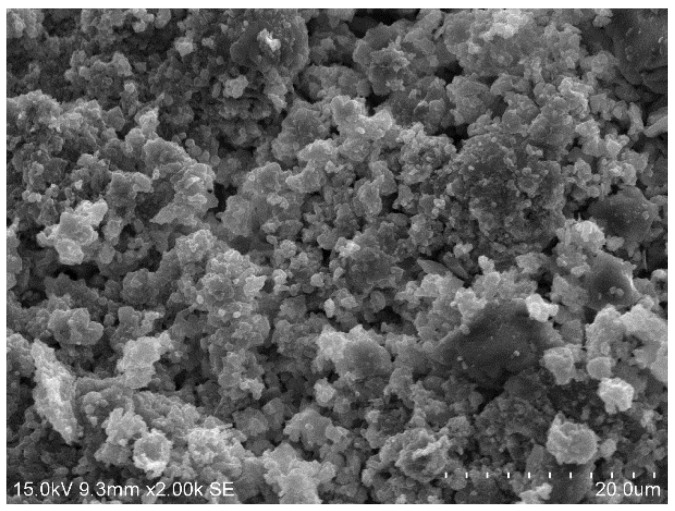
2000× SEM image of the A_1_BC_2_ combination.

**Figure 12:**
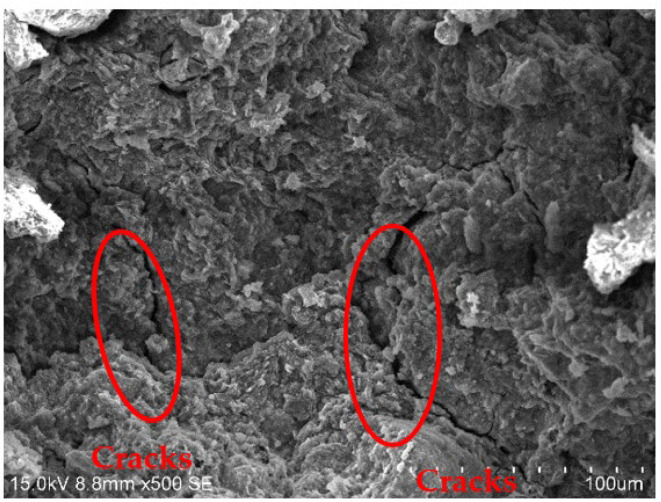
500× SEM image of the A_3_BC_2_ combination.

**Figure 13:**
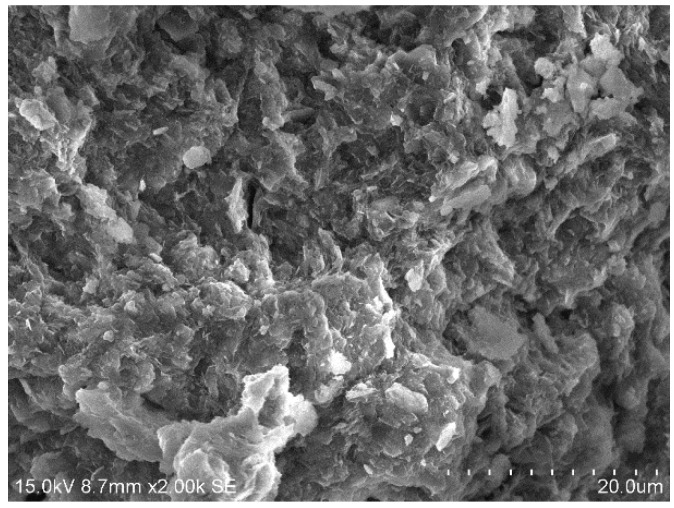
2000× SEM image of the A_3_BC_2_ combination.

Figure 10, Figure 11, Figure 12 and Figure 13 show the scanning electron microscopy results of the A_1_BC_2_ and A_3_BC_2_ specimens cured for 7 days. Different microstructures of the A_1_BC_2_ and A_3_BC_2_ combinations were observed. Regarding the A_1_BC_2_ specimen that was mainly composed of red mud, Figure 10 shows that the surface of the specimen was compact and continuous without cracks and cavities. The compact structure of the specimen was considered to provide this good stress-bearing capacity. Figure 11 shows that the red mud masses and the surrounding pores were filled with white fine particles without obvious holes or cracks, while on the A_3_BC_2_ specimen mainly composed of tailings mud, cracks can be identified clearly in Figure 12. These cracks were causing damage to the integrity of the specimen, thus worsening the stress-bearing capacity. Figure 13 shows that the surface of the specimen was uneven but without obvious holes or cracks (Figure 12).

The content of Na_2_O in the red mud reached 9.21%, which enhanced the alkali environment of the specimen during the condensation and hardening process, making the A_1_BC_2_ combination stronger. The kaolinite content of the tailings mud reached 37.9% [25], which made the tailings mud itself have a limited swelling and shrinkage ability, resulting in swelling and shrinkage cracking of the solidified body (Figure 13), affecting the structural integrity of the specimen. Therefore, the strength of the A_3_BC_2_ combination is lower than that of the A_1_BC_2_ combination.

**Figure 14:**
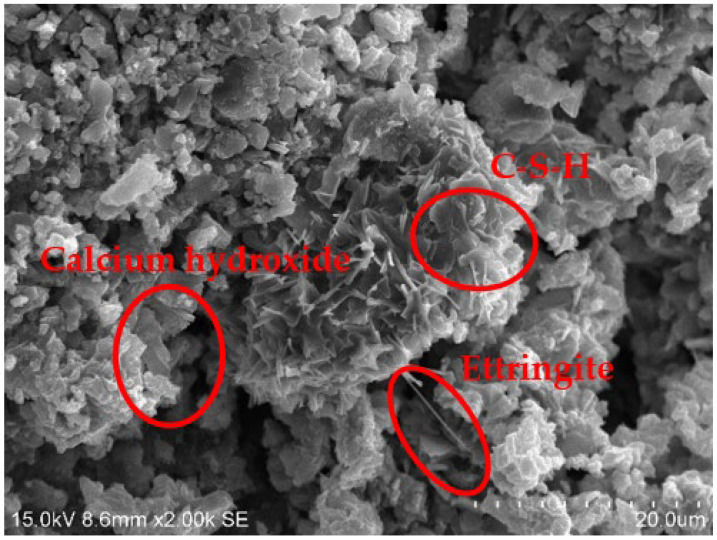
Image of the hydration products of the A_1_BC_2_ Combination (1).

**Figure 15:**
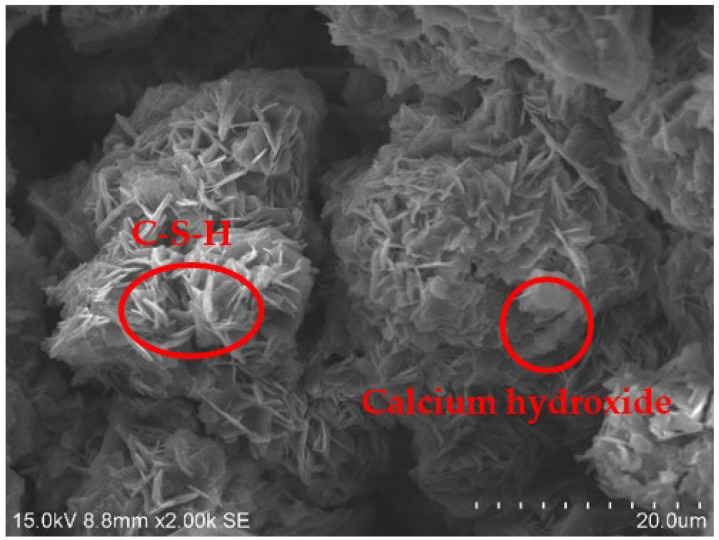
Image of the hydration products of the A_1_BC_2_ Combination (2).

Furthermore, the specimens were compacted to a block structure. In such structures, the particles of the mixture were closely connected. From this point of view, the mixture’s initial strength is attributed to its own friction force. Then, with the increase in curing time, the chemical reaction between the mixed materials occurs when cement materials encounter water. The amorphous crystal hydrated calcium silicate (C–S–H), cube or flake calcium hydroxide, and needle flake ettringite were generated (Figure 14 and Figure 15). The quick lime also reacted with water to form calcium hydroxide. In the alkaline environment, Ca^2+^ ions released by cement and lime reacted with Al_2_O_3_ and SiO_2_ that were rich in wastes, causing a pozzolanic reaction. New products such as calcium silicate hydrate and calcium aluminate hydrate were formed [26,27]. The main reaction equations are shown in Equations (1) and (2).

Ca(OH)_2_ + SiO_2_ + nH_2_O → CaO·SiO_2_·(n + 1) H_2_O (1)

Ca(OH)_2_ + Al_2_O_3_ + nH_2_O → CaO·Al_2_O_3_·(n + 1) H_2_O (2)

With age, hydration reactions and pozzolanic reactions continued to produce hydration products. Therefore, the cementitious material can not only bond to the mixture particles but also fill the voids in the structure, thus forming a network structure, reducing structural porosity [28], improving the compactness of the structure, and eventually forming a subgrade water-stable material with higher strength.

### 3.5. pH Tests

**Figure 16:**
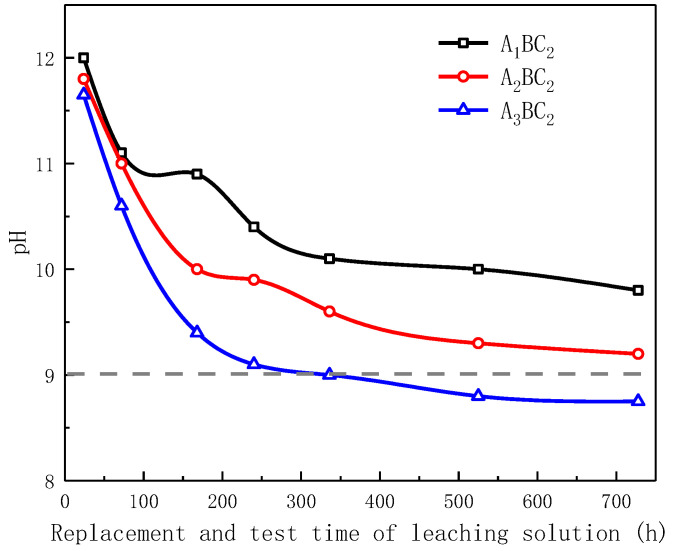
Variation of pH values with leaching time.

The pH tests were carried out on the leaching solution of the A_1_BC_2_, A_2_BC_2_ and A_3_BC_2_ combination specimens cured for 28 days. The replacement test time of the leaching solution was 1 d, 3 d, 7 d, 10 d, 14 d, 21 d, and 28 d. The test results are shown in Figure 16. The pH values of the leaching solutions range from 8.75 to 12. With the increase in the leaching time, the pH value showed a decreasing trend. The pH values of the first three leaching solutions decreased rapidly and then decreased slowly. When the replacement test time of the leaching solution is 28 d, the pH values of the leaching solutions of A_1_BC_2_, A_2_BC_2_, and A_3_BC_2_ are 9.8, 9.2, and 8.75, respectively. When the replacement test time of the A_3_BC_2_ combination leachate was 7 days, the pH value had dropped to 9. The reason for the decrease in the pH value of this leaching solution is summarized as: (i) the cementitious products generated by hydration reactions and pozzolanic reactions solidified Na^+^ ions and inhibited the release of OH^—^, and (ii) the addition of tailings mud reduced the amount of red mud, that is, reduced the content of Na_2_O in raw materials. According to the Chinese standard GB3838-2002 [29], a pH value of 9 is the limit for surface water environmental quality; the final pH value of the leaching solution of the A_3_BC_2_ combination does not exceed the limit of surface water environmental quality. To this end, its environmental compatibility is the best.

## 4. Conclusions

In this experiment, red mud and bauxite tailings mud were used to develop subgrade material, and the compressive strength of the material was verified to meet the requirements of subgrade. From the experimental results, four conclusions can be drawn as follows:

(1) The UCS of all of the combinations increased with curing time. The UCS of the specimen with a ratio of red mud to tailings mud of 2:1 was higher than that of the specimen with a ratio of 1:2. With the same ratio of red mud to tailings mud, the UCS with a ratio of waste to cementitious materials of 1:0.2 was the highest, followed by that with a C of 1:0.1, and that with a C of 1:0.3 was the lowest. The UCS of the A_1_BC_2_, A_2_BC_2,_ and A_3_BC_2_ combinations exceed 2 MPa in 7 days, and the CBR is higher than 6% in 7 days, which meets the strength requirements of class II and below low-grade highways. Among them, the combination with the highest strength is the A_1_BC_2_ combination: the UCS was 3.03 MPa in 7 days, and UCS was 4.54 MPa in 28 days.

(2) Microstructural investigation showed that the A_1_BC_2_ combination with more red mud than tailings mud had a compact and continuous structure without cracks, leading to good stress-bearing capacity. However, cracks were observed in the A_3_BC_2_ combination with more tailings mud than red mud. This difference in microstructures leads to an obvious difference in UCS. It shows that the mechanical properties of red mud as subgrade material are better than that of tailings mud.

(3) After curing for 28 days, the final pH values of the experimental leaching solutions of the A_1_BC_2_, A_2_BC_2_, and A_3_BC_2_ composite samples were 9.8, 9.2, and 8.75, respectively. The cementitious products produced by hydration and pozzolanic reactions solidified Na^+^ ions and inhibited the release of OH^−^. Meanwhile, the higher the amount of tailings mud was in the mixture, the lower the amount of Na_2_O was in the mixture, which also promoted decreases in the pH values of tested subgrade materials. The A_3_BC_2_ composite sample has the best environmental compatibility.

(4) The testing results prove the feasibility of the use of red mud and tailings mud in subgrade materials: these two aluminum industrial wastes can be reused. A feasible scheme for the consumption of red mud and tailings mud was proposed. Moreover, cement and lime are commonly used cementitious materials. It is more economical to use part of lime instead of cement.

## Figures and Tables

**Table 1:** Main chemical components of red mud and tailings mud (%).

Chemical Components	Fe_2_O_3_	Al_2_O_3_	SiO_2_	CaO	Na_2_O	K_2_O	MgO	TiO_2_
red mud	29.5	21.6	15.1	11.5	9.21	0.16	0.60	5.59
tailings mud	15.43	38.02	27.62	0.37	/	0.75	/	1.6

**Table 2:** Combination scheme for mixtures of waste muds and cementitious materials.

Number	Mass Ratio of Red Mud and Tailings Mud	Mass Ratio of Cement and Quicklime	Mass Ratio of Waste and Cementitious Materials	Mixture ID
1	A_1_	B	C_1_	A_1_BC_1_
2	A_1_	B	C_2_	A_1_BC_2_
3	A_1_	B	C_3_	A_1_BC_3_
4	A_2_	B	C_1_	A_2_BC_1_
5	A_2_	B	C_2_	A_2_BC_2_
6	A_2_	B	C_3_	A_2_BC_3_
7	A_3_	B	C_1_	A_3_BC_1_
8	A_3_	B	C_2_	A_3_BC_2_
9	A_3_	B	C_3_	A_3_BC_3_

**Table 3:** Specifications of the testing program.

Type of Test	Tested Mixture	Initial Water Content of Specimen (%)	Dimensions of Specimen (mm)	Curing Duration (Days)	Curing Conditions
Compaction test	all mixture listed in Table 2	17–40	Φ 100 × H 127	0	/
UCS test	all mixture listed in Table 2	optimum water content	Φ 50 × H 50	1, 7, 28, 60	20 °C, 95% humidity
	BC_2_ combinations	optimum water content	Φ 50 × H 50	28	20 °C, immersion in water
CBR test	A_3_BC_2_	optimum water content	Φ 50 × H 50	7	20 °C, 95% humidity

## Data Availability

Some or all data, models, or code that support the findings of this study are available from the corresponding author upon reasonable request.
